# *HSP90* Controls *SIR2* Mediated Gene Silencing

**DOI:** 10.1371/journal.pone.0023406

**Published:** 2011-08-04

**Authors:** Shyamasree Laskar, Mrinal K. Bhattacharyya, Rama Shankar, Sunanda Bhattacharyya

**Affiliations:** 1 Department of Biotechnology, School of Life Sciences, University of Hyderabad, Hyderabad, Andhra Pradesh, India; 2 Department of Biochemistry, School of Life Sciences, University of Hyderabad, Hyderabad, Andhra Pradesh, India; Texas A&M University, United States of America

## Abstract

In recent years, Hsp90 is found to interact with several telomeric proteins at various phases of cell cycle. The Hsp90 chaperone system controls assembly and disassembly of telomere structures and thus maintains the dynamic state of telomere. Here, for the first time we report that the activity of another telomeric protein Sir2p is modulated by Hsp82, the ortholog of Hsp90 from budding yeast (*Saccharomyces cerevisiae*). In a temperature sensitive Hsp90 deficient yeast strain (*iG170Dhsp82*), less abundant Sir2p is observed, resulting in de-repression of telomere silencing and a complete loss of mating type silencing. Intriguingly, over expression of Hsp90, either by exposing cells to heat shock or by introducing *HSP82* overexpression plasmid also yields reduced level of Sir2p, with a consequential loss of telomere silencing. Thus, Hsp90 homeostasis maintains the cellular pool of Sir2p and thereby controls the reversible nature of telomere silencing. Interestingly, such regulation is independent of one of its major co-chaperones Sba1 (human ortholog of p23).

## Introduction

Hsp90 is a highly abundant eukaryotic protein, which is involved in maturation and folding of some special class of proteins, which are primarily involved in signal transduction [1], [2], [3]. It dimerises in an ATP dependent manner with several cochaperones and provides the maturation of the target protein at a near native state [4]. In budding yeast there are two isoforms of Hsp90; Hsc82 (human ortholog of Hsp90β), which is constitutively expressed in the cell and Hsp82 (human ortholog of Hsp90α), which is induced whenever cells are exposed to any kind of stressed condition. It is known that expression of either one of the two isoforms of Hsp90 is required for yeast viability [5]. The two isoforms share 97% sequence identity and they comprise (1–2) % of the total cytosolic proteins. Hsp90 level is significantly increased in the cell upon exposure to stress, including temperature, nonphysiological pH, nutrient deprivation and malignancy [6]. Recent studies have unraveled novel roles of Hsp90, where Hsp90 and its cochaperone p23 are involved in stabilization of different protein DNA complexes during RNA transcription, telomere maintenance, DNA replication *etc.* [7], [8], [9], [10]. Hsp90 and p23 also influence *in vitro* and *in vivo* telomerase activity [11]. It is also known that other cochaperones, for example, Hsp70, Hsp40 and Hop are also needed for proper maturation of telomerase [12]. However, unlike the steroid hormone receptor maturation, where Hsp90-p23 complex is only transiently attached to the target protein, in the case of telomerase activation, Hsp90 and p23 are found to be associated with functional telomerase complex even after the completion of telomere addition. In yeast, Hsp82 (the ortholog of Hsp90) helps in maintaining a dynamic equilibrium between the extendable and unextendable state of telomere through active interaction with Cdc13 [13]. In recent years, a plethora of proteomic, bioinformatics, and genetic approaches unravel many more telomeric proteins as potential clients of Hsp82. Examples include Cdc13, Stn1, [14], [15], Sir2 [16], Mre11 [17], Ku80, Mec1 and Est1 [15]. Some of these gene products are involved in telomere position effect (TPE) [18], a phenomenon where transcription of gene is repressed by its close proximity to telomere.

In budding yeast, telomere silencing is initiated by recruitment of Sir2p-Sir4p complex at subtelomeric regions by telomere bound Rap1p and Ku70/Ku80 heterodimer. Sir2p mediated histone deacetylation helps Sir3p binding, which triggers the spreading of the Sir complex along the subtelomeric regions, causing heterochromatinisation. Sir2p, which is the key member of sirtuins, is conserved across the evolution [19]. It is a NAD^+^ dependent histone deacetylase and is involved in transcriptional silencing of the silent mating type loci, *HML* and *HMR*, as well as genes near telomeres [20], [21]. A high throughput study aims at identifying *S. cerevisiae* deletion mutants affecting telomere length reveal slightly short telomere phenotype in *hsc82* deletion mutant [22]. However contradictory results from other studies show no apparent change in telomere length in *hsp82* or *hsc82* deletion mutants [14]. It is well established that length of telomere positively regulates the efficiency of silencing, *i.e.*, the longer the telomere, greater is the silencing [23]. However, the effect of Hsp82 deficiency on TPE has not been explored. Moreover, why over-expression of Hsp82 leads to telomere length shortening is also poorly understood, except for the fact that *HSP82* and *HSC82* are the high copy suppressors for the *stn1-1* and *cdc13-1* mutants [14].

In this paper, we show that Hsp82 homeostasis plays a critical role in maintaining cellular pools of enzymatically active Sir2p. In *Δhsp82*, *Δhsc82* double knock out background, which is maintained by *iG170Dhsp82* temperature sensitive allele, Sir2p level is found to be considerably diminished which is accompanied by complete loss of Sir2p function at restrictive temperature. Our result shows that at restrictive temperature, both the mating type silencing as well as telomere silencing function is completely lost, a phenotype similar to that observed in *Δsir2* cells. This conclusively proves that Sir2p function is dependent on Hsp82. On the other hand, over-expression of Hsp82 shows negative regulatory effect on telomere silencing but has no effect on mating type silencing. Our result shows that Hsp82 over expression diminishes the level of Sir2p. We hereby speculate that under normal physiological condition, Hsp82 stabilizes Sir2p and thereby maintains repressed state of subtelomeric chromatin. On the other hand, under stressed condition, which results during over-expression of *HSP82*, the level of Sir2p is down regulated that de-represses the silent chromatin state. Thus, it seems that the central role of Hsp82 is to maintain a dynamic equilibrium of the level of silencing proteins which may be responsible for the reversible nature of telomere silencing.

## Materials and Methods

### Yeast strains

Strains used in this study are listed in Table 1. The strain SLY20 used for TPE color assay is isogenic to W303a having *ADE2* marked telomere VIIL. The mating tester strains used for these studies are YDS31 and YDS32. The temperature sensitive strain *iG170Dhsp82* was kindly provided by Didier Picard [24]. This strain has the permissive temperature 25°C and restrictive temperature (35°C–37°C). Yeast knock out strains YSC1021-551935 (*HSP8*2*::KAN* ^r^), YSC1021-552834 (*SBA1::KAN* ^r^), YSC1021-551520 (*HSC82::KAN* ^r^) were purchased from Thermo Scientific, Open Biosystems, Huntsville, AL, USA. All primers used in this study were purchased from Sigma Aldrich. Using the genomic DNA of YSC1021-551520 as a template and the pair of primers OSB1 (5′ACC AAG CGT TGG GTA ATG A3′) and OSB2 (5′ TGG TCA TTT GAC AGC TGA TG3′) KANMX cassette with *HSC82* flanking regions was amplified. The PCR product was then integrated into SLY20 and selected on G418 sulphate containing plates. The resultant *hsc82* null strain is referred as SLY4 in this paper. In a similar way, using genomic DNA of YSC1021-551935 (*HSP82::KAN* ^r^) as a template and the pair of primers OSB3 (5′ TGA CAC ACT AGA CGC GTC GG3′) and OSB4 (5′TAC CAA CCA GGT CCT TCC GC3′) KANMX cassette with *HSP82* flanking regions was amplified. It was then integrated to SLY20 to generate *hsp82* null strain SLY5. To knock out SBA1 gene, the genomic DNA of YSC1021-552834 was used as a template to amplify KANMX cassette with *sba1* flanking regions. Forward primer OSB5 (5′ TGC TAC CCG CCT TCC GAG TG3′) and the reverse primer OSB6 (5′ CAC ATA CAG TTC CAT TAC TTG AC3′) were used for this purpose. The PCR product was then integrated into SLY20 cells to generate *sba1* null strain SLY6.

**Table 1:** Yeast strains used in this study.

Strains	Genotype
SLY20	*MATa 15ade2-1, ura3-1, 112 his 3-11, trp1, leu2-3, VIIL::ADE2*
YDS31	*MATa his1^−^*
YDS32	*MATα his1^−^*
*iG170Dhsp82*	*MATa can1-100 ade2-1 his3-11,15 leu2-3,112 trp1-1 ura3-1 HSP82::LEU2* *HSC82::LEU2 HIS3::HSP82G170D*
YSC1021-551520	*MATa HSC82::KAN* ^r^
YSC1021-551935	*MATa HSP82::KAN* ^r^
YSC1021-552834	*MATa SBA1::KAN* ^r^
SLY4	*MATa 15ade2-1, ura3-1, 112 his 3-11, trp1, leu2-3, VIIL::ADE2 HSC82::KAN* ^r^
SLY5	*MATa 15ade2-1, ura3-1, 112 his 3-11, trp1, leu2-3, VIIL::ADE2 HSP82::KAN^r^*
SLY6	*MATa 15ade2-1, ura3-1, 112 his 3-11, trp1, leu2-3, VIIL::ADE2 SBA1::KAN* ^r^
SLY12	*MATa 15ade2-1, ura3-1, 112 his 3-11, trp1, leu2-3, VIIL::ADE2 SIR2::KAN* ^r^
SLY13	*MATa 15ade2-1, ura3-1, 112 his 3-11, trp1, leu2-3, VIIL::ADE2 pHCA/HSP82*
SLY10	*MATa 15ade2-1, ura3-1, 112 his 3-11, trp1, leu2-3, VIIL::ADE2 HSP82::KAN^r^* *pHCA/HSP82*
SLY31	*MATa 15ade2-1, ura3-1, 112 his 3-11, trp1, leu2-3, VIIL::ADE2 SBA1::KAN^r^* *pHCA/HSP82*
SLY32	*MATa 15ade2-1, ura3-1, 112 his 3-11, trp1, leu2-3, VIIL::ADE2 pTA/HSP82*
SLY46	*MATa 15ade2-1, ura3-1, 112 his 3-11, trp1, leu2-3, VIIL::ADE2 pHCA*

The *sir2* null strain SLY12 (*sir2::KAN^r^*) was made by integration of KANMX cassette with *SIR2* flanking regions in SLY20. All of the knockout genotypes were confirmed by PCR analysis.

The *HSP82* over expression plasmid pHCA/hsp82, which has *HIS3* marker, was transformed into SLY20, SLY5 and SLY6 strains and the transformed colonies were selected by growing them on SC-his medium to derive SLY13, SLY10 and SLY31 strains respectively. The blank vector pHCA was transformed into SLY20 and the transformed colonies were selected on SC-his medium to derive the strain SLY46. The 2 µ *HSP82* over expression plasmid pTA/hsp82 which has *TRP* marker was transformed into SLY20 strain; the transformed colonies were selected by growing them on SC-trp medium to derive SLY32 strain.

### Plasmids

pHCA/*HSP82* plasmid was a gift from Didier Picard [25]. It is a CEN/ARS plasmid, derived from pRS313 vector, which over expresses *HSP82* under the control of GPD promoter.

Using genomic DNA of W303a as a template we amplified the *HSP82* gene using the forward primer OSB21 (5′ GAC GGA TCC ATG GCT AGT GAA ACT TTT GAA TTT C 3′) having *Bam*H1 site and the reverse primer OSB22 (5′ CGG GTC GAC CTA ATC TAC CTC TTC CAT TTC GG 3′) having *Sal*I sequence. The PCR amplified product was then cloned into *Bam*H1, *Sal*I double digested 2 µ expression vector pTA, which expresses *HSP82* under the control of GPD promoter. All the constructed plasmids were confirmed by sequencing.

### TPE color assay

SLY20, SLY4, SLY5, SLY6, SLY13, SLY12, SLY31 and SLY46 cells were grown on appropriate medium and TPE was performed according to the protocol described [26].

### Mating type frequency assessment

Wild type strain (*HSP82*), *Δhsp82*, *Δhsc82*, *YDS32 (MATα)* and *Δsir2* were grown to the mid exponential phase. About 400 cells from each culture were plated in YPD plate and incubated at 30°C. Also 400 cells of the wild type (*HSP82*), *Δhsp82*, *Δhsc82*, and *Δsir2* were mixed with same number of cells of *YDS32 (MATα)* and incubated at 30°C shaker for 10 minutes. Then each of them was spreaded on SD (synthetic dextrose) plate and incubated at 30°C incubator for 30 hours. The number of cells grown on each YPD and SD plates were counted. The mating frequency was calculated as = (No. of cells grown on SD plate/No. of cells on YPD plate)* 100. The value obtained in case of *Δsir2* strain was subtracted from each of the three strains. The experiment was repeated for 5 times and each bar shows the mean frequency ± SD.

### Western blotting

The temperature sensitive strain *iG170Dhsp82* was grown at 25°C (permissive temperature) as well as 37°C (restrictive temperature) and protein was isolated from them by the procedure described below. Proteins were isolated from SLY4, SLY5, SLY6, SLY10, SLY13, SLY20 and SLY32 strains after growing them on proper medium at 30°C and equal amounts of proteins were loaded in SDS PAGE. For heat shock treatment, wild type cells (SLY20) were grown upto 0.3 O.D. at 595 nm. It was then divided into two batches, one batch of cells was subjected to heat shock at 39°C for 40 minutes, and other was grown at 30°C. Equal amount of cells were finally harvested and protein was isolated from them.

We isolated the protein using trichloroacetic acid (TCA) as described in [27] with little modifications. Each of the above strains was grown on 20 ml of appropriate medium until the OD_600 nm_ reaches 0.5. The cell was centrifuged and the pellet was washed first with distilled water and then with 500 µl 20% TCA. The pellet was finally resuspended in 200 µl 20% TCA and the cell lysis was done with glass beads keeping at cold temperature for 30 min. The TCA precipitated proteins were collected; washed with 5% TCA and dissolved in 60 µl 1× sample buffer (0.05 M Tris-HCl [pH 6.8], 2% sodium dodecyl sulfate [SDS], 10% glycerol and 0.1% bromophenol blue), supplemented with 6.66 µl 1 M DTT, and 33 µl 1 M Tris HCl, [pH 9.0]. The protein samples were boiled for 3 minutes and appropriate volumes of samples were loaded on 10% SDS poly acrylamide gel. The gel was then transferred to a polyvinylidene difluoride (PVDF) membrane and immuno blotted for Hsp82, Sir2 and Actin. The anti Hsp90 antibody (Calbiochem), the anti Sir2 (Santa Cruz Biotechnology Inc., CA) and anti Actin antibody (Abcam) were used at 1∶5000 dilution. Horseradish peroxide-conjugated rabbit IgG (Santa Cruz Biotechnology Inc., CA) was used as a secondary antibody for Sir2 at 1∶10000 dilution and HRP conjugated mouse IgG (Promega) was used as a secondary antibody for Hsp82 and Actin blot at the same dilution. The Western blots were developed using chemiluminescent detection system (Pierce). The bands on the gel were quantified using Gene Tools, Syngene and the relative densities, thus obtained, were plotted using GraphPad Prism 5 software. The mean value from four independent experiments was plotted with standard deviations (±SD). All blots were normalized against Actin.

### RNA isolation

Total RNA was isolated from *iG170Dhsp82* after growing them at 25°C and 37°C, as well as from SLY20, SLY12 and SLY13 strains by using acid phenol method as described [28] with some modifications. 10 ml culture of cells were grown up to mid log phase (OD_600_≈1), centrifuged and the pellet was suspended in 400 µl TES buffer (10 mM Tris HCl, [pH = 7.5], 10 mM EDTA, 0.5% SDS). 400 µl phenol (pre equilibrated with water) was added and the mixture was incubated at 650C for 1 hour, with intermittent vortexing. The mixture was rapidly chilled on ice for 5 min, and centrifuged at 14000 rpm for 10 min at 40C. The aqueous layer was mixed with 400 µl chloroform, vortexed and centrifuged again for the same time at the same speed and temperature mentioned above. The extracted aqueous phase was precipitated by adding 1/10^th^ volume of 3 M sodium acetate [pH 5.2] and 2.2 volume of chilled ethanol. The precipitate containing RNA was washed with 70% ethanol and the pellet was dissolved in 30 µl DEPC treated water. Equal amount of RNA measured by spectroscopic analysis (JASCO spectrophotometer EMC-709) was then subjected to DNase I (Fermentas) digestion for 15 min at room temperature. DNase I was finally inactivated by incubating with 25 mM EDTA at 650C for 10 min. About 10 µg of total RNA from each sample was then reverse transcribed with oligo dT primer (Sigma Aldrich) using reverse transcriptase (Omniscript kit, Qiagen). Resulting cDNA was first quantified, diluted appropriately to normalize and then subjected to PCR amplification (27 cycles) using gene specific primers. The PCR products were run on 1.4% agarose gel and stained with ethidium bromide. We used OSB16 (5′TGA CCA AAC TAC TTA CAA CTC C3′) and OSB14 (5′TTA GAA ACA CTT GTG GTG AAC G3′) for amplifying 307 base pair at the 3′ end of *ACT1* transcript. OSB19 (5′ ATC ACG AGT AAG GAT CAA AG 3′) and OSB20 (5′ TTA TGG CTT TGT TAC GCT TG 3′) were used to amplify *YFR057w*, which is located in the subtelomeric region on chromosome VIR. We used OSB62 (5′ AAT CGG CGG ATG GGT TGG 3′) and OSB63 (5′ TCA TTC TTT CTT CTT TGC CAG 3′) for amplifying 308 base pair at the 3′end of the *HMLα2* transcript.

## Results

### Sir2p function is dependent on Hsp82

**Figure 1:**
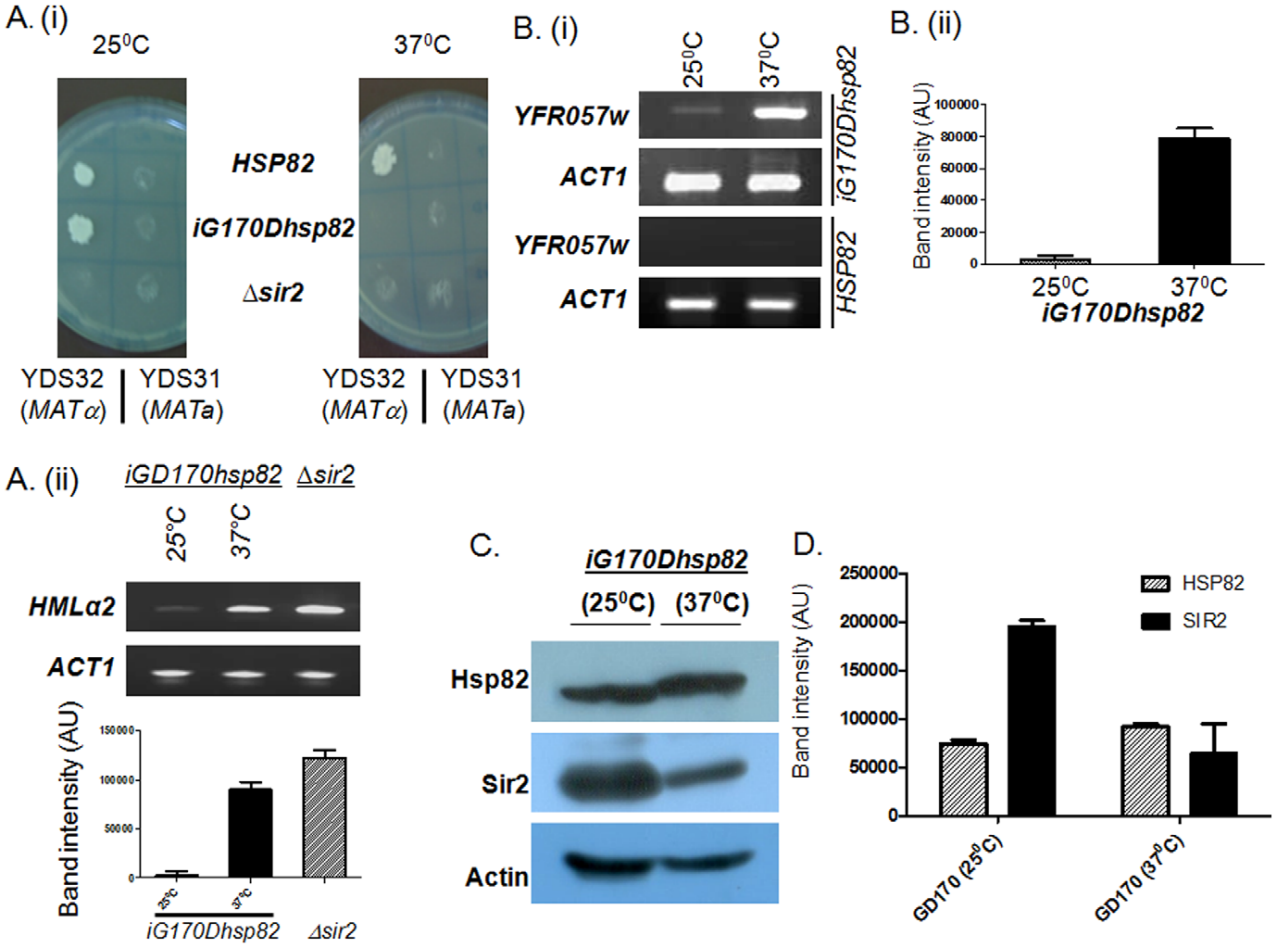
Sir2p function is dependent on Hsp82. (Ai) The temperature sensitive strain *iGD170hsp82* causes disruption of mating type silencing at restrictive temperature (37°C), where Hsp82 is non functional. (Aii) *iG170Dhsp82* at 37°C shows considerable amount of *HMLα2* transcript comparable to that present in *Δsir2* cells; whereas at 25°C negligible amount of *HMLα2* is seen. (Bi) Semi quantitative RT-PCR shows increase in *YFR057w* transcript in *iG170Dhsp82* at 37°C compared to 25°C, indicating that the strain has defect in telomere position effect, whereas wild type strain (*HSP82*) shows silencing at higher temperature (37°C). (Bii) Graphical representation of four independent experiments of (Bi) is done after normalization with *ACT1* control. (C) Western blot shows the relative abundance of Hsp82 and Sir2p at two temperatures. Actin is the loading control. (D) The quantification of Western blot (from 4 independent experiments) shows more than 50% reduction in steady state level of Sir2p at higher temperature. The data are normalized with respect to the loading control Actin. Each bar represents mean density ± SD.

Previously, yeast two hybrid screen, using Hsp82 as bait, has identified Sir2p interaction [16]. To explore whether Sir2p function is dependent on Hsp82, we examined both the mating type silencing as well as telomere position effect in a conditional mutant, where Hsp82 is functionally defective. We performed the assay using a temperature sensitive *iG170Dhsp82* strain. In this strain both *HSP82* and *HSC82* genes are deleted and the strain behaves as wild type when grown at permissive temperature 25°C, but as mutant when grown at restrictive temperature at 37°C [24]. This particular strain harbors mutant *G170Dhsp82* which is integrated into its chromosome. It tolerates little bit higher temperature than its counterpart where *G170Dhsp82* is maintained episomaly in the cell [29]. Previous work by Nathan and Lindquist showed that the mutant *G170Dhsp82* when present as an episomal copy within the cell, its growth is significantly diminished above 34°C. However if it is integrated within the chromosome it can tolerate (2–3)°C higher temperature [24], [30]. We allowed to mate *iG170Dhsp82* (*MATa*) with tester strain YDS32 (*MATα*) at 25°C and 37°C and compared its mating behavior with wild type (*HSP82*) and *Δsir2* strains. We found that loss of function of *HSP82* at 37°C impaired the mating ability of the strain and it behaved like *Δsir2* (Figure 1Ai). Conversely, at 25°C, the strain showed mating ability comparable to wild type cells. In order to rule out the possibility that the lack of silencing at restrictive temperature is not due to any lack of growth, we tested the viability of both temperature sensitive mutant and that of the tester strains at 37°C, and found that all the strains were viable at 37°C (Figure S1).

It was earlier reported that Hsp90 controls the pheromone signaling in yeast [25]. Therefore, it is important to understand whether the loss of mating type silencing of *G170Dhsp82* at restrictive temperature is due to the defect in pheromone signaling pathway alone, or also due to de-repression of *HMLα* locus. To address this question the semi quantitative RT-PCR was done to measure the *α2* transcript level from *G170Dhsp82 (MATa)* after growing them at 25°C as well as 37°C (Figure 1Aii). The quantification RT-PCR data showed negligible amounts of *α2* transcript at 25°C, whereas at 37°C significant amount of *α2* transcript was visible. This result was well corroborated with the sterility of *iG170Dhsp82* at 37°C, since in order for a *MATa* strain to behave as a mating compatible haploid strain, *α2* factor should remain repressed [31], [32]. As a control, we had taken *Δsir2* strain where the RT-PCR result showed the presence of considerable amount of *α2* transcript, which made it sterile. Thus from this experiment it can be concluded that in *hsp82* deficient condition, there is a loss of silencing at HML, which is not only due to defect in pheromone signaling pathway [25] but also due to derepression of *MATα2* transcript in *iGD170hsp82 (MATa)*.

We also explored whether Hsp82 has any role in silencing of subtelomeric genes. For that purpose we monitored the mRNA level of *YFR057w* gene, which is located adjacent to chromosome VIR telomere. *YFR057w* ORF codes for a protein of unknown function and is located within 1 kb from the end of chromosome. As Sir2p spreads as far as 3 kb length in the chromosome VIR [33], its ORF is repressed under normal conditions but de-repressed in *Δsir2* strain (Figure S2). We quantified the steady state level of *YFR057w* transcript in *iG170Dhsp82* at both 25°C and 37°C and compared with *ACT1* transcript. The semi quantitative RT-PCR showed significant increase in *YFR057w* transcript at 37°C compared to 25°C, whereas the level of *ACT1* transcript remained the same at both temperatures (Figure 1B). In order to rule out the possibility that the loss of silencing is not a mere effect due to shift in temperature we measured the *YFR057w* transcript level of wild type strain (SLY20) at both 25°C and 37° (Figure 1B). No loss of silencing was observed in wild type strain at higher temperature. This result is consistent with the previous finding that higher temperature causes more silencing [34]. The above two experiments conclusively prove that Hsp82 has a role in regulating telomere silencing as well as mating type silencing.

Since, Sir2p is the key protein involved in both of these silencing mechanisms; we wanted to see whether there is any change in the level of Sir2p in *iGD170hsp82* strain at different temperatures by Western blot analysis. When monitored at non permissive temperature (37°C) the level of Sir2p was found to be substantially diminished (Figure 1C), whereas the level of Hsp82 was comparable at both the temperatures, Act1p being a loading control. The experiment was repeated four times and the mean value of the quantification of the band intensity on Western blot showed that there was at least 50% reduction in the level of Sir2p at 37°C compared to 25°C (Figure 1D). We hereby speculate that Sir2p may be a putative client of Hsp82, whose activity inside the cell is directly or indirectly regulated by Hsp82. At restrictive temperature when Hsp82 is nonfunctional, Sir2p level is reduced by 50% and thus at lower levels it is unable to provide mating type silencing and telomere silencing activity. Thus for transcriptional silencing at telomere and at mating type loci, the budding yeast requires native Hsp82 protein.

### Effect of Hsp82 over-expression on mating type silencing and transcriptional silencing at telomere

**Figure 2:**
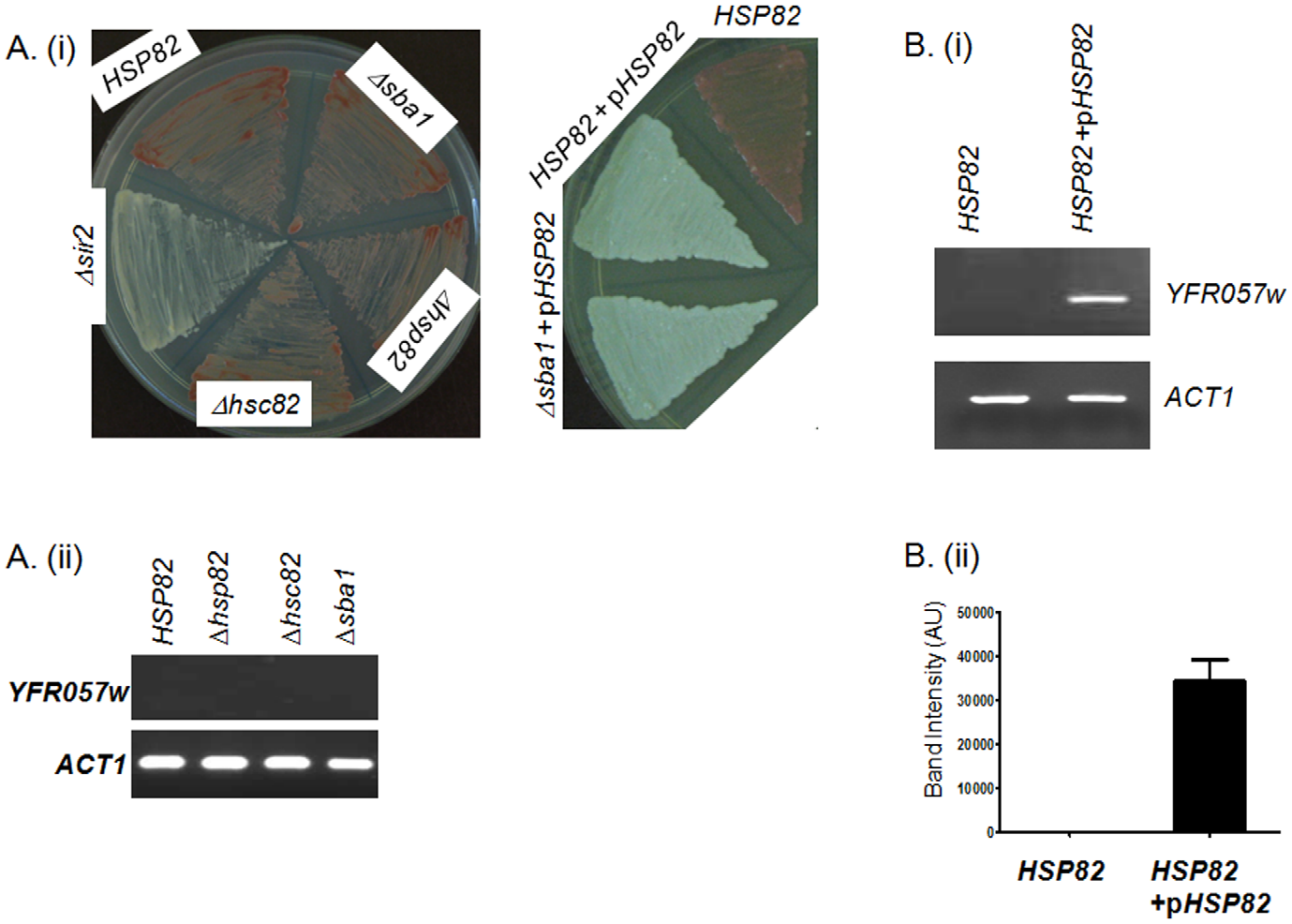
Over-expression of *HSP82* negatively regulates TPE but not mating type silencing. (Ai) ADE2 color phenotype of various strains *i.e.*, *HSP82* (wild type), *Δhsp82*, *Δhsc82*, *Δsba1*, *Δsir2*, over-expressing *HSP82* (*HSP82+pHSP82*) and over-expressing *HSP82* in *Δsba1* background. Pink colored colony represents telomere silencing and white colored colony represents de-repression of telomere silencing. (Aii) *YFR057w* is silenced in all single mutants as well as in wild type strain. (Bi) Over-expression of *HSP82* de-represses the expression of *YFR057w*. *ACT1* serves as loading control. (Bii) Quantification of *YFR057w* transcript is done from 4 independent experiments in *HSP82* overexpressed cells. The band intensity values are normalized against *ACT1*. Each bar represents mean density ± SD.

Next, we wanted to determine whether single knockout mutant allele *Δhsp82* or *Δhsc82* has any effect on Sir2p function. We generated two deletion mutants, *hsp82* and *hsc82* in SLY20 strain, which had *ADE2* gene next to telomere at chromosome VIIL. Under normal condition, when SLY20 was allowed to grow on low adenine medium at 30°C, they developed mostly as red colored colonies due to the lack of *ADE2* expression, whereas *Δsir2* null strain SLY12 developed as white colored colonies owing to the expression of *ADE2*. Our result showed that deletion mutants SLY4 (*Δhsc82*), SLY5 (*Δhsp82*) exhibited wild type phenotype, *i.e*. they all produced pink colored colony (Figure 2Ai) due to transcriptional silencing of *ADE2* gene, suggesting that Hsp82 and Hsc82 are redundant to each other. Similarly when we checked *YFR057w* transcript level of the single deletion mutants, we found *YFR057w* was silenced in all of them as expected from earlier experiment (Figure 2Aii). Thus single knockout alleles do not show any change in transcriptional silencing at subtelomeric locus and are redundant to each other.

As Hsp82 level is increased within the cells whenever cells are exposed to any kind of stressed conditions, we aimed to determine the effect of elevated amount of Hsp82 on Sir2p level and examined whether the silencing function of Sir2p was altered in Hsp82 over-expressed condition. We mimicked the Hsp82 over-expressed condition within the cell by transforming a centromeric plasmid pHCA/hsp82 in SLY20 to generate SLY13 strain, where Hsp82 was expressed under a strong promoter GPD. Interestingly, we found SLY13 cells resulted mostly in white colored colonies indicating a decrease in TPE (Figure 2Ai), however SLY46 strain containing the blank vector showed pink color colonies. In order to test whether the loss of TPE, due to over-expression of *HSP82*, is restricted to chromosome VII alone or other subtelomeric genes are also de-repressed, we monitored the mRNA level of *YFR057w* gene. We quantified the steady state level of *YFR057w* transcript in Hsp82 over-expressed cell (SLY13) and compared with wild type cell (SLY20). The semi quantitative RT-PCR showed significant increase in *YFR057w* transcript in Hsp82 over-expressed cells compared to the wild type, whereas *ACT1* transcript remained same in both the genotype (Figure 2B). This leads us to speculate that over-expression of Hsp82 acts as a negative regulator of transcriptional silencing at telomeres.

**Figure 3:**
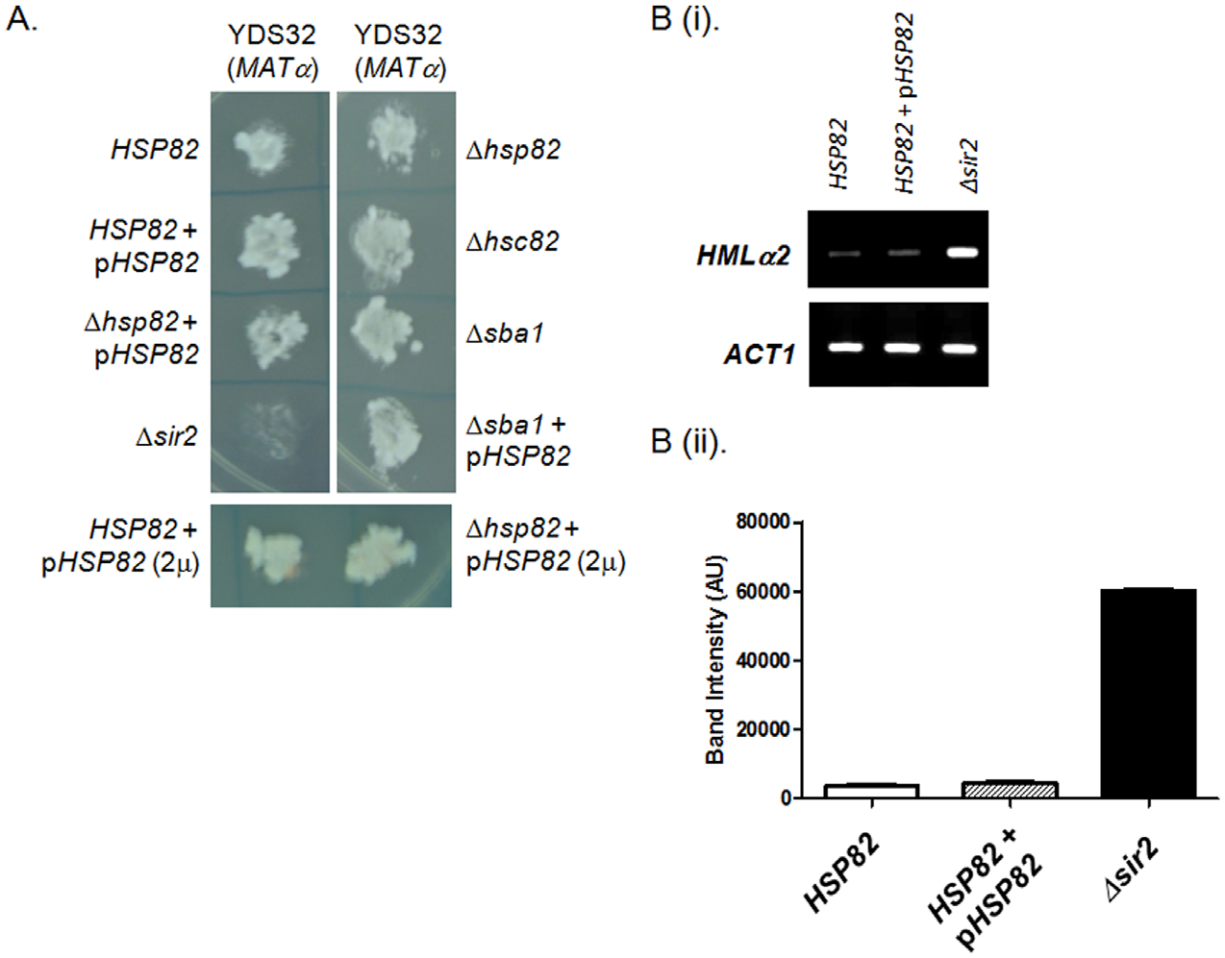
Single mutants as well as overexpression of *HSP82* do not alter mating type silencing. (A) Mating type silencing phenotype of different strains (as indicated) when mated with the tester strain YDS32 (*MATα*). *Δsir2* acts as a negative control. (Bi) RT-PCR analysis of *HMLα2* transcripts in different strain backgrounds (as indicated) shows that *HMLα2* transcript levels are similar in *HSP82* overexpressing cells and wild type cells, *ACT1* serves as loading control. (Bii) Quantification of *HMLα2* transcript from three independent experiments are normalized with *ACT1* and plotted. Each bar represents mean density ± SD.

In order to find out the effect of single knock out (*Δhsp82* or *Δhsc82*) strains or Hsp82 over-expression on mating type silencing, we measured the mating ability of SLY4, SLY5, SLY10, SLY13, and SLY32 and compared with wild type and *Δsir2* strains. It was observed that all of them exhibited wild type mating phenotype when mated with the tester strain YDS32 (*MATα*) (Figure 3A). The mating type frequency for each of the deletion mutants (*Δhsp82* and *Δhsc82*) were plotted (Figure S3) and it was found to be comparable with that of the wild type. We also determined the level of *MATα2* transcript in *HSP82* overexpressing cell (SLY13) and compared its level with wild type (SLY20) and *Δsir2* (SLY12) strains. While *Δsir2* strain showed abundant levels of *MATα2* as expected, SLY13 showed negligible amount of *MATα2* transcript comparable to that of the wild type (Figure 3B). We, therefore, conclude that neither the single knockout mutants nor the over-expression of Hsp82 affect mating type silencing.

We were also interested to explore whether Sba1 (human ortholog of p23), which is a cochaperone of Hsp82 has any role in Sir2p maturation. To this end we created *Δsba1* deletion strain SLY6. We had also over-expressed Hsp82 in *sba1* null background (SLY31). While doing TPE assay for both SLY6 and SLY31, we found that SLY6 mostly developed as pink colored colonies whereas SLY31 showed white colored colonies (Figure 2Ai). The *Δsba1* deletion strain could also silence the subtelomeric gene *YFR057w* (Figure 2Aii) like wild type. The mating type silencing function of both SLY6 and SLY31 remained unperturbed (Figure 3A). These results indicate that Hsp82 mediated homeostasis of Sir2p is independent of Sba1.

### Over-expression of Hsp82 reduces the cellular pool of Sir2p in a dose dependent manner

**Figure 4:**
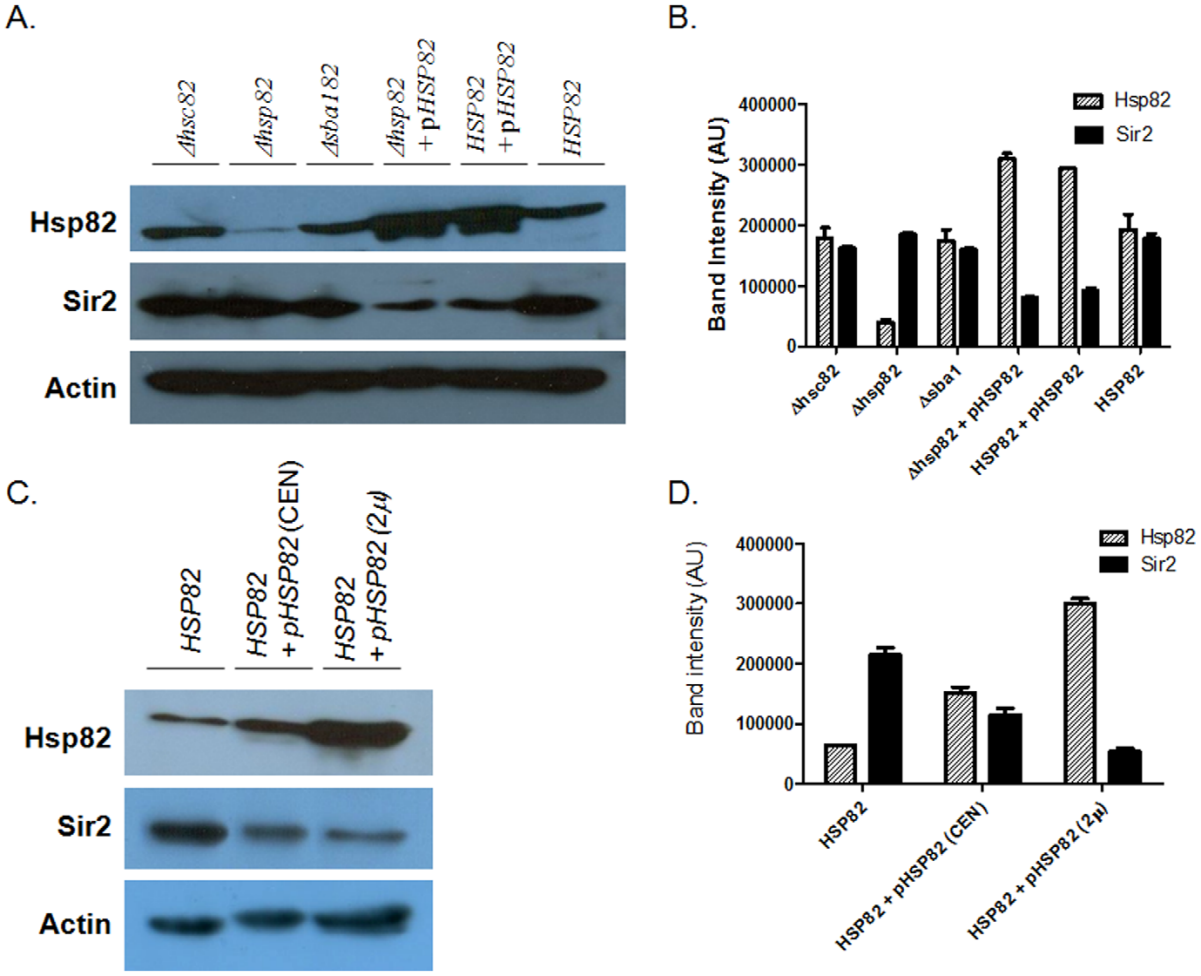
Western blot analysis shows over-expression of *HSP82* down regulates Sir2p in a dose dependent manner. (A) Different lanes are marked with the respective genotypes. p*HSP82* implies over-expression of *HSP82* from a CEN plasmid. Total proteins from each lysates are probed with anti Hsp82 antibody, anti-Sir2 antibody and with anti-Actin antibody (as loading control). (B) Graphical representation of (A), where densitometric measurements of the bands from four experiments are plotted, after normalizing with Actin band intensities. Each bar represents mean density ± SD. (C) Multiple levels of Hsp82 over-expression is shown using high (2 µ) and low copy plasmids (CEN), to demonstrate dose response between Hsp82 and Sir2p levels. (D) The graphical representation of Western blot as in figure (C) shows over expression of Hsp82 from a high copy plasmid confers more reduction of Sir2p level. Each bar represents the mean density ± SD from 4 independent experiments. The data are normalized with respect to Actin.

As over-expression of Hsp82 leads to down regulation of transcriptional silencing at telomeres, we monitored whether there was any change in Sir2p level as a function of Hsp82 expression. Equal amount of proteins isolated from different strains was loaded and they were probed with anti-Hsp90 antibody as well as anti-Sir2 antibody. Our result showed that in Hsp82 over-expressing cells (SLY10 and SLY13 strains) the level of Sir2p was considerable diminished (Figure 4A). The quantification of the bands from at least three independent experiments showed that in SLY13 strain while Hsp82 amount was increased by 50% compared to the wild type, the level of Sir2 was reduced by more than 50% in some cases (Figure 4B). In all other strain background such as SLY4 (*Δhsc82*), SLY5 (*Δhsp82*), SLY6 (*Δsba1*) the level of Sir2p remained unaltered. These results also suggest that Hsc82 (which is poorly detected by the antibody) compensates for the lack of Hsp82 (in SLY5) and thus the level of Sir2p is not affected.

Since cellular level of Hsp82 changes in response to different stressed condition, it is important to know whether Sir2p level is dependent on Hsp82 in a dose dependent manner or not. In order to address this question, we generated another strain SLY32, which over-expresses Hsp82 from a multicopy 2 µ plasmid (pTA/hsp82). We compared the level of Hsp82 and Sir2 among SLY20 (endogenous *HSP82*), SLY13 (endogenous plus one copy from a CEN plasmid) and SLY32 (multiple copies of *HSP82*) strains by Western blot analysis. Our result showed a gradual increment in Hsp82 protein levels from SLY20 to SLY13 to SLY32, which was in accordance with the copy number. However, the level of Sir2p showed a reverse trend. The most dramatic reduction in Sir2p level was observed in SLY32 cells, while SLY13 exhibited a moderate decrement compared to the wild type cells (SLY20) (Figure 4C). The quantification of the bands, taking the average from independent three sets of experiment, showed that while the increase in Hsp82 level is 4 times in SLY32 strain compared to the wild type, the decrease in Sir2 level was at least by 75% compared to the wild type and by 50% compared to the SLY13 strain (Figure 4D). Thus, it appears that under stressed condition the increased level of Hsp82 results in concomitant reduction of the total cellular pool of Sir2p, which begs us to speculate that such adaptation in Sir2p abundance in response to Hsp82 homeostasis could be responsible for the reversible nature of transcriptional silencing at telomere.

### Heat shock induces derepression of telomere silencing in yeast

**Figure 5:**
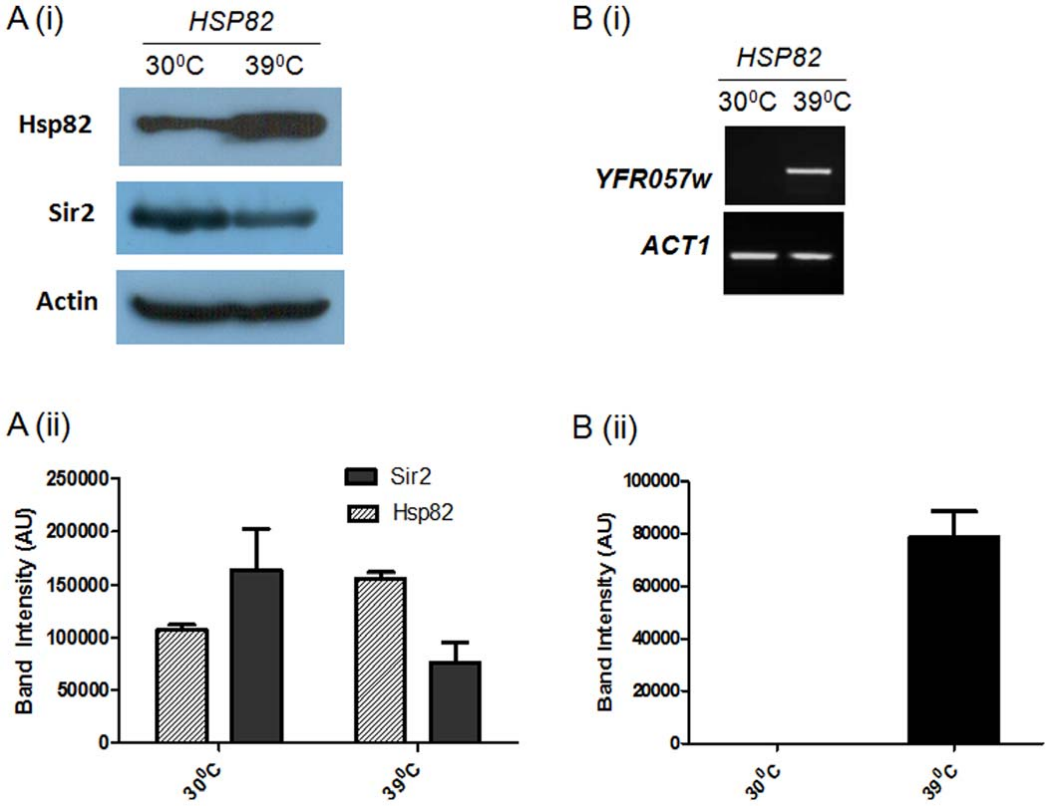
Heat shock induces derepression of transcriptional silencing at telomere. (Ai) The Western blot shows the relative levels of Hsp82p, Sir2 and Act1 in wild type strain at normal *vs*. heat shock condition. (Aii) Three independent experimental data are normalized with respect to Actin and plotted. Each bar represents mean density ± SD. (Bi) Heat shock induces the derepression of subtelomeric gene *YFR057w*, *ACT1* being the loading control. (Bii) Three independent experiments are performed and the data are plotted after normalizing with *ACT1*. Each bar represents mean density ± SD.

Our findings propose that the Sir2p level and/or their function is controlled by Hsp82 homeostasis, which may be responsible for reversible nature of telomere silencing in yeast. This observation led us to think whether heat shock induced elevated level of Hsp82p is sufficient to alter the Sir2p level and consequently its function. To achieve this we exposed wild type cells (SLY20) to 39°C heat shock for 40 minutes. This is the optimum condition for induction of heat shock gene *HSP82* [35]. After heat shock we harvested the cell and isolated protein from them. The Western blot analysis showed (Figure 5Ai) similar kind of reduction in Sir2p level in heat stressed condition compared to cells growing at 30°C. The experimental data were plotted (Figure 5Aii) which showed heat shock induced the expression of Hsp82p which in turn reduced about 50% of the total cellular pool of Sir2p.

In order to find out the effect of heat shock on telomere silencing we aimed to monitor the transcript level of the subtelomeric gene *YFR057w*. For that we isolated the total RNA from the wild type cells grown under normal (30°C) as well as stressed condition (exposing at 39°C for 40 min). The semi-quantitative RTPCR showed the abundance of *YFR057w* transcript as a result of heat shock, indicating loss of telomere silencing (Figure 5B). These data strengthen our model that Hsp82 homeostasis is indeed responsible for the reversible nature of transcriptional silencing at telomere. Though the mechanism of this is not clear at present, but it is evident that Hsp82 is directly or indirectly controlling the cellular abundance of Sir2p.

## Discussion

Several important findings came out through this study. Firstly, our study shows for the first time that Sir2p function is directly or indirectly dependent on Hsp82, and its steady state level in the cell is also controlled by Hsp82. We observe that in *iGD170hsp82* temperature sensitive mutant, where Hsp82 is nonfunctional at restrictive temperature, there is significant reduction of Sir2p which is associated with loss of mating type silencing as well transcriptional silencing at telomere. As Hsp82 controls pheromone signaling pathway in yeast, we wanted to decipher whether the loss of mating type silencing at restrictive temperature is also due to derepression at the *HMLα* locus in *MATa* strain. Our experimental result shows significant amount of *MATα2* transcript at 37°C, which is well correlated with the sterile nature of *iG170Dhsp82 (MATa)*. The Western blot shows that considerable amount of Sir2p is still present at the restrictive temperature (albeit at 50% reduced level); however functional studies at hidden mating locus demonstrated that these Sir2 proteins are inadequate for its functional activity. It has been reported earlier that silencing in yeast is dependent on temperature [34]. Increase in the temperature is associated with stronger TPE and mating type silencing in yeast. As an explanation of this effect it has been shown that at higher temperature Sir2-Net1 complex is destabilized whereas Sir2-Sir4 complex is having no effect on temperature [36]. Thus at higher temperature more Sir2 is available to bind with Sir4 leading to an increase in telomere and mating type silencing at the expense of ribosomal silencing in yeast. In our system the derepression of transcriptional silencing both at telomere and *MAT* locus at higher temperature is directly linked with the lack of Hsp82/Hsc82 activity.

Secondly, over-expression of Hsp82 in the cell causes reduction of Sir2p level in a dose dependent manner. This has important consequence as occasionally the amount of Hsp82 increases within the cell when they are exposed to heat shock or subjected to other stressed condition. Our study demonstrates, in a condition where Hsp82 is overexpressed, there occurs a gradual decrement of Sir2p which impairs its ability in transcriptional silencing at telomere, although silencing at *HML*/*HMR* locus remains unperturbed. This data suggests that under stressed condition, though Sir2p level is diminished, there are enough Sir2p still present to perform other cellular activities. We speculate that although Sir2p is the key protein to control both mating type silencing and telomere silencing, the threshold cellular concentration of Sir2p for these two different functions may be different. In case of silencing at the mating type loci, the reduced cellular level of Sir2p, which is the consequence of Hsp82 over-expression, may be sufficient to maintain the silence state, however, it may be limiting for silencing the stretch of subtelomeric regions spread over 32 chromosomal ends. The question remains that why at low concentration, Sir2p comes out of the subtelomeric region, but not from the mating type loci. Considering that telomere silencing is a reversible phenomenon, meaning that the silencing complex is in a dynamic equilibrium between DNA bound and unbound states; and mating type silencing is not reversible under normal conditions, provoke us to speculate that the binding affinity of silencing complex towards the silencer sequences at the cryptic mating type loci is much stronger than that for the subtelomeric regions. Thus, binding of Sir proteins at the silent mating loci may be achieved at much less cellular concentration.

It is important to understand how deficiencies of Hsp82 as well as overexpression of Hsp82 both lead to derepression of TPE. Our results suggest that under overexpression condition the loss of TPE activity is due to the reduced cellular pool of Sir2p; on the other hand in *Δhsp82Δhsc82* double mutant cell (*iG170Dhsp82*) the derepression of silencing is not only due to reduced level of Sir2p but could also be due to the presence of functionally inactive Sir2p. It appears that even the low abundance of active Sir2p (as low as 1/4^th^ of the usual amount) is good enough to ensure mating type silencing (as observed in SLY32 cells). The absence of *MATα2* transcript in *HSP82* overexpressing cells also confirms their mating ability. We reason that these cells are capable of mating type silencing, because they still possess wild type *HSC82* and *HSP82* genes due to which Sir2p maturation and functional activation are perhaps unaltered. Taking all these together, it is very tempting to propose that loss of TPE under overexpression condition and knockout condition could be mechanistically distinct. At present we do not know whether Hsp82 is directly controlling the cellular pool of Sir2p or it does so through a mediator.

Thirdly, our result suggests that Hsp82 over-expression leads to euchromatinisation of chromosome ends. The transitions between the two states of chromatin are very well studied in TPE model in budding yeast *S. cerevisiae* [18]. However, the reversible nature of such heterochromatin-euchromatin transition and *vice versa* is poorly understood. Although, the epigenetic marks (especially H3 and H4 modifications) associated with such changes are well established [37], the regulation at the DNA-protein interface is ill understood. It is known to a greater extent that how do the silencing proteins nucleate and then spread along the heterochromatin [38]. However, the molecular events occurring during the exit of the silencing complex from the heterochromatin giving rise to euchromatin formation is largely unknown. Findings from this work leads us to speculate that may be under *in vivo* condition Hsp82 is required to maintain a dynamic equilibrium of total amount of silencing proteins. This idea has been proven under stressed conditions such as heat shock that alter Hsp82 levels in the cells. We have exposed the cells to heat shock and under that conditions observed that total Sir2p abundance is also reduced by more than 50% which is associated with complete lose of silencing at telomere. Thus the adaptation in Sir2p abundance in response to the Hsp82 homeostasis could be responsible for the reversible nature of transcriptional silencing at telomere. We speculate that Hsp82 modulates interaction of silencing complex (Sir2-Sir3-Sir4) with DNA in such a way that it facilitates the removal (or degradation) of the major silencing proteins, once the chromatin does not need to be maintained at the heterochromatin state. Similar effect is also observed at telomere end, where Hsp82 acts as a releasing factor for Cdc13 to control the telomere protein dynamics, and maintains the extendable and unextentable states of telomere [13].

Finally, our genetic analysis shows that Hsp82 mediated maturation of Sir2 is independent of its cochaperone Sba1. This observation does not fit with classical steroid hormone receptor chaperone model, where Sba1 is found to be required at the last stages during client release. Thus, apart from traditional chaperonic role, newer roles of Hsp82 are emerging, where it acts as a regulator of various DNA protein interactions occurring inside the cell. Our report also provides evidence along the same line. Future studies need to be done to understand how Hsp82 actually regulates Sir2 level.

## Supporting Information

Figure S1 **Temperature sensitivity of** ***iG170Dhsp82*** **.** (A) Growth kinetics of *iG170Dhsp82* at indicated temperatures. This strain shows slow growth phenotype at 37°C. (B) *iG170Dhsp82* cells, wild type cells (*HSP82*), tester strains YDS32 (*MATα*) and YDS31 (*MATa*) all show comparable growth at 37°C on YPD plate.
(TIF)

Figure S2 ***Δsir2*** **strain shows de-repression of telomere silencing at** ***YFR057w*** **locus.** Semi quantitative RT-PCR shows increase in *YFR057w* transcript in *Δsir2* strain compared to the wild type strain (*SIR2*). *ACT1* transcript level remains comparable in both the strains.
(TIF)

Figure S3 **Mating type frequency of wild type,** ***Δhsp82*** **and** ***Δhsc82*** **.** Wild type (*HSP82*), *Δhsp82* and *Δhsc82* mating type frequencies show comparable values.
(TIF)
