# Supplementary figures and images for: HSP90 Controls SIR2 Mediated Gene Silencing
Source: PLoS One. 2011 Aug 4;6(8):e23406. doi: 10.1371/journal.pone.0023406 (PMC3150437; doi:10.1371/journal.pone.0023406)

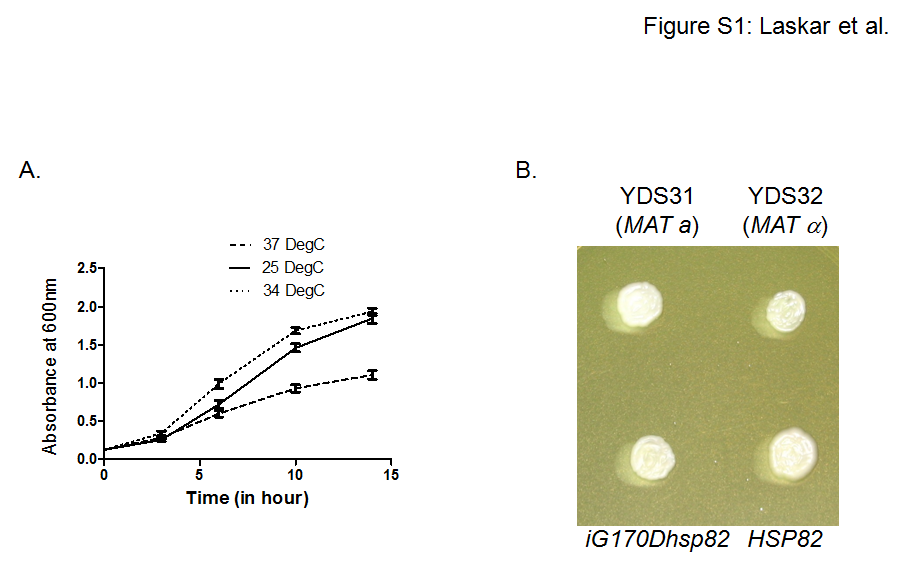

Supplement: Figure S1 — Temperature sensitivity of iG170Dhsp82 . (A) Growth kinetics of iG170Dhsp82 at indicated temperatures. This strain shows slow growth phenotype at 37°C. (B) iG170Dhsp82 cells, wild type cells (HSP82), tester strains YDS32 (MATα) and YDS31 (MATa) all show comparable growth at 37°C on YPD plate. (TIF) [file pone.0023406.s001.tif]

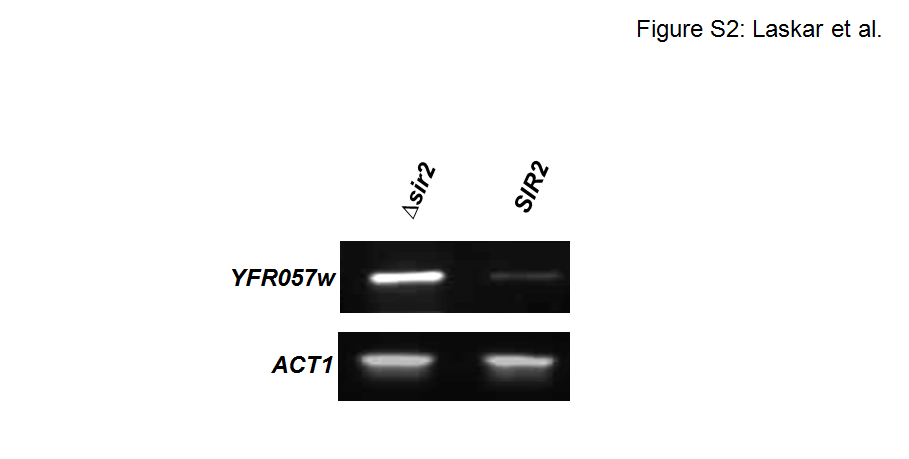

Supplement: Figure S2 — Δsir2 strain shows de-repression of telomere silencing at YFR057w locus. Semi quantitative RT-PCR shows increase in YFR057w transcript in Δsir2 strain compared to the wild type strain (SIR2). ACT1 transcript level remains comparable in both the strains. (TIF) [file pone.0023406.s002.tif]

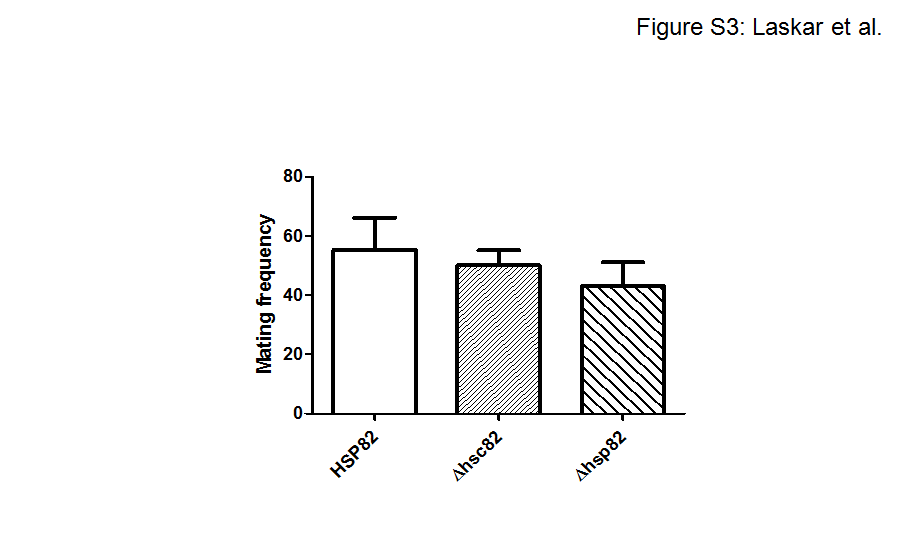

Supplement: Figure S3 — Mating type frequency of wild type, Δhsp82 and Δhsc82 . Wild type (HSP82), Δhsp82 and Δhsc82 mating type frequencies show comparable values. (TIF) [file pone.0023406.s003.tif]
